# Development of an Accurate Mass Retention Time Database for Untargeted Metabolomic Analysis and Its Application to Plasma and Urine Pediatric Samples

**DOI:** 10.3390/molecules26144256

**Published:** 2021-07-13

**Authors:** Chiara Lavarello, Sebastiano Barco, Martina Bartolucci, Isabella Panfoli, Emanuele Magi, Gino Tripodi, Andrea Petretto, Giuliana Cangemi

**Affiliations:** 1 Core Facilities-Clinical Proteomics and Metabolomics, IRCCS Istituto Giannina Gaslini, 16147 Genoa, Italy; ChiaraLavarello@gaslini.org (C.L.); MartinaBartolucci@gaslini.org (M.B.); 2 Department of Chemistry and Industrial Chemistry, University of Genoa, 16146 Genoa, Italy; emanuele.magi@unige.it; 3 Chromatography and Mass Spectrometry Section, Central Laboratory of Analyses, IRCCS Istituto Giannina Gaslini, 16147 Genoa, Italy; SebastianoBarco@gaslini.org (S.B.); GinoTripodi@gaslini.org (G.T.); GiulianaCangemi@gaslini.org (G.C.); 4 DIFAR-Biochemistry Laboratory, University of Genoa, 16132 Genova, Italy; Isabella.Panfoli@unige.it

**Keywords:** LC-HRMS, metabolomics, library, chromatography, pediatrics

## Abstract

Liquid-chromatography coupled to high resolution mass spectrometry (LC-HRMS) is currently the method of choice for untargeted metabolomic analysis. The availability of established protocols to achieve a high confidence identification of metabolites is crucial. The aim of this work is to describe the workflow that we have applied to build an Accurate Mass Retention Time (AMRT) database using a commercial metabolite library of standards. LC-HRMS analysis was carried out using a Vanquish Horizon UHPLC system coupled to a Q-Exactive Plus Hybrid Quadrupole-Orbitrap Mass Spectrometer (Thermo Fisher Scientific, Milan, Italy). The fragmentation spectra, obtained with 12 collision energies, were acquired for each metabolite, in both polarities, through flow injection analysis. Several chromatographic conditions were tested to obtain a protocol that yielded stable retention times. The adopted chromatographic protocol included a gradient separation using a reversed phase (Waters Acquity BEH C18) and a HILIC (Waters Acquity BEH Amide) column. An AMRT database of 518 compounds was obtained and tested on real plasma and urine samples analyzed in data-dependent acquisition mode. Our AMRT library allowed a level 1 identification, according to the Metabolomics Standards Initiative, of 132 and 124 metabolites in human pediatric plasma and urine samples, respectively. This library represents a starting point for future metabolomic studies in pediatric settings.

## 1. Introduction

Metabolomics is an emerging technology that allows the comprehensive study of the low molecular weight molecules within an organism [1,2]. It represents a powerful tool for precision medicine, being helpful for understanding the mechanisms of diseases, in the discovery of new therapeutic targets or biomarkers for diagnosis and monitoring the activity of therapeutics [3,4,5]. High resolution mass spectrometry (HRMS) is an increasingly used instrument for metabolomics, thanks to its high performance allowing the analysis of complex matrices and the detection of hundreds of metabolites [6]. One of the major bottlenecks of metabolomics is the identification process of compounds that is necessary to draw any biological conclusion from untargeted metabolomics data [7]. Data processing workflows incorporate several defined steps, such as noise filtering, peak detection, peak deconvolution, retention time alignment and finally feature annotation [8]. Feature annotation is performed by comparing an experimental mass measurement to a database of known metabolites in order to generate potential candidates. Several databases (such as mzCloud, HMDB, METLIN or MassBank) were developed to assist researchers in compound putative identification and are continuously implemented by the scientific community [9,10,11]. Moreover, given the wide variety of chromatographic and mass spectrometric conditions that can be employed to generate metabolomic experiments, it is essential that established metabolomics laboratories implement fixed, robust and reliable protocols that yield stable retention times [12].

Standardization in metabolomics is still an unmet need but many efforts have been made towards realizing this aim. In 2007, the Metabolomics Standards Initiative (MSI) defined the guidelines for reporting the minimum metadata relative to metabolite identification with four different levels with level 1 being the highest confident identification when a structure is confirmed with a minimum of two independent and orthogonal data from a pure reference standard under identical analytical conditions [13].

The aim of this paper is to describe the development of an Accurate Mass Retention Time (AMRT) database starting from a commercial metabolite library of standards (MSMLS). This database has been integrated into the protocol used in our laboratory for metabolomic untargeted analyses and was tested on human plasma and urine samples derived from pediatric subjects.

## 2. Results

### 2.1. Accurate Mass Retention Time Library

**Figure 1:**
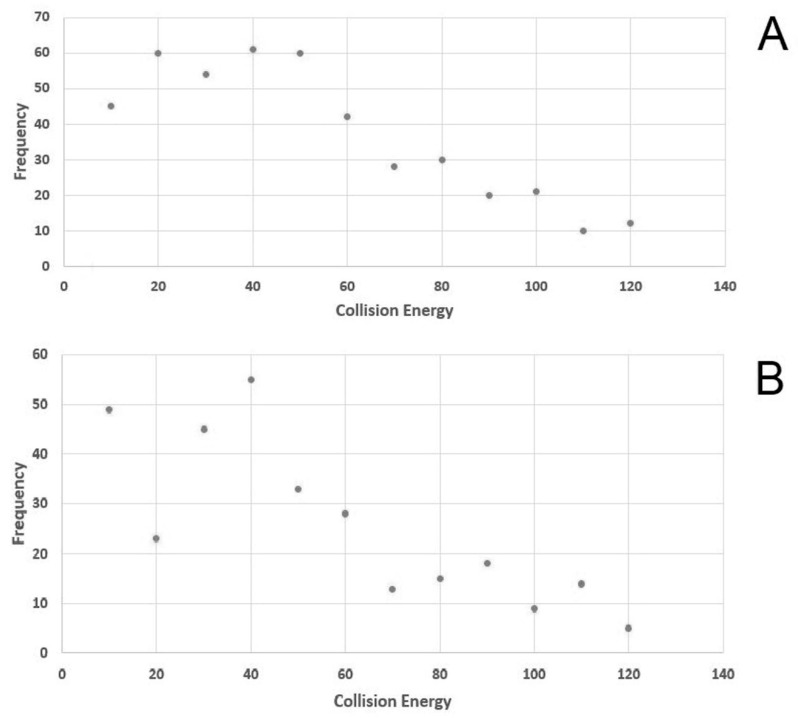
Figure shows in ordinates the number of molecules and in abscises the best normalized collision energy. The panel above (**A**) is show positive mode results and in panel below (**B**) in negative mode.

An AMRT library was obtained using a commercial metabolite library of standards (MSMLS) composed by 634 metabolites (listed in Supplementary Table S1). Each molecule was individually characterized through flow injection analysis (FIA). The MS^1^ (molecular ion or adduct) was selected in both negative and positive polarity and fragmented using 12 different normalized collision energies (NCE) (a parameter that is adopted by Thermofisher Scientific instruments) in order to collect a complete and sharable database that might be also used with other mass spectrometers. Optimal NCE with a high degree of confidence in the identification of compounds and structural information in our setting were selected. The library of the list of compounds, the molecular ions and the fragmentation spectra were shared as a public format (file format *.msp). Figure 1 schematizes NCE adopted and shows that the most frequently used are 20, 40 and 50 in positive mode and 10, 30, 40 in negative mode.

The chromatographic conditions which allowed the best results in terms of peak suitability score to be obtained, in our hands, were the following: ACQUITY BEH C18 (with mobile phase A: 0.1% formic acid in H_2_O and mobile phase B 0.1% formic acid in acetonitrile) for reversed phase and ACQUITY BEH Amide (with mobile phase A: 5 mM ammonium formate in H_2_O pH 3 and mobile phase B 100% acetonitrile) for hydrophilic interaction liquid chromatography (HILIC) separation. The choice of reversed-phase column was quite straightforward as, for both polarities, ACQUITY BEH C18 fulfilled both threshold values (e.g., symmetry and width values—50 positive mode and 67 negative mode, Table 1). For HILIC columns the choice was more difficult: at pH 8, two columns showed good performances, with a value of 32 for both Accucore Amide HILIC and Shodex Asahipak NH2P-50 2D, but at pH 2 the same columns showed the worst performances. Therefore, we decided to use the ACQUITY BEH Amide column because it maintained a better performance on both polarities (Table 1).

A total of 359 and 191 compounds were identified by using reversed phase and HILIC columns, respectively. Supplementary Table S2 shows the AMRT database obtained containing the list of compounds with their optimal NCE and the corresponding RT.

### 2.2. Analysis on Real Samples

**Figure 2:**
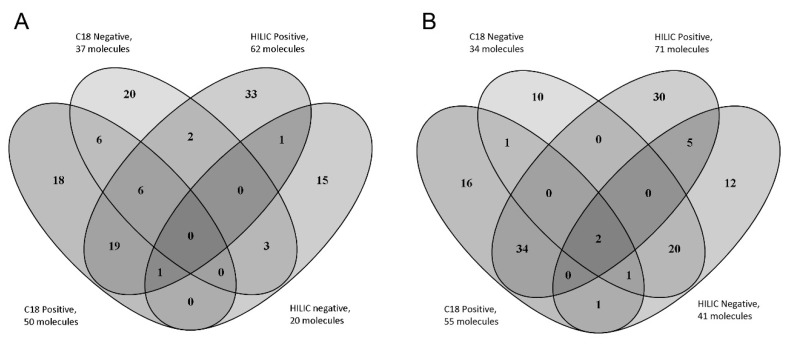
Metabolomic analysis of plasma (**A**) and urine (**B**) pool samples. Venn Diagram of the total number of level 1 identified metabolites in real samples using reversed phase and HILIC columns both in positive and negative polarities.

A total of 132 and 124 compounds (listed in Supplementary Table S3) belonging to the AMRT database were identified with level 1 identification in plasma and urine samples, respectively. Figure 2 shows the number of metabolites identified in plasma and highlights the importance of combining the different chromatographic conditions and MS polarities in order to increase the number of identified compounds [14]. The identified metabolites were preliminarily checked in order to verify their concordance with the expected results. Some key metabolites, consistent with the pediatric origin of our study samples, could be identified both in plasma and urine samples: betaine (associated to low adiposity), acetyl-carnitine (typically increased after starvation), indoxyl sulphate (deriving from hepatic transformation of indole, in turn a microbiome tryptophan (Trp) metabolite). Interestingly, two other Trp metabolites synthesized by gut microbiota, indole-3-methyl acetate and 5-Hydroxyindoleacetic acid, were found in urine samples [15,16,17,18].

Moreover, a number of metabolites associated with diet components were identified. Oxalic and ascorbic acid, two metabolites associated with a diet rich in fruits and vegetables were found in plasma; creatinine, associated with a diet rich in proteins was found both in plasma and in urine. P-hydroxyphenylacetic acid and riboflavin, associated with a diet rich in nuts and seeds and dairy products, respectively, were identified in urine samples [19]. Some metabolites related to carbohydrates (3-hydroxybutyric, citric, and aconitic acid), amino acids (proline, valine, leucine, isoleucine) and lipids (suberic acid) were also identified in urine. All these findings are in line with a Mediterranean diet (MD), consistent with the origin of our study subjects and in line with the findings of a previously published study on 1H NMR human urinary metabolome profiles of individuals following an MD [20]. Moreover, the metabolites identified in urine indicating a low-fat diet, such as hippurate, histidine and its derivates methyl-histidine, carnosine, and anserine, were also identified [20].

## 3. Discussion

Untargeted metabolomic analysis is a powerful technique, in that it allows the collection of data without pre-existing knowledge, being, thus, very useful for several applications [21]. Sample preparation protocols, chromatographic conditions and instrument platforms have an impact on the results directly influencing the subset of metabolites that can be found. One of the bottlenecks of the untargeted strategy is represented by the correct identification of the molecules which represents a crucial point for drawing the correct biological conclusions [7].

A few published papers describe similar findings but, differently from ours, the resulting spectral libraries and RT databases were not shared in a public format [22,23].

The retention time database that we share is strictly related to our chromatographic conditions, so it can be useful only for those who decide to strictly adopt our choice of columns, phases and flow rates. Whereas some variables, such as dead volumes, specific to the system used are barely controllable, we suggest using our data as a starting point. This approach allowed us to obtain a level 1 identification of about 400 metabolites that can also be integrated in small molecule identification software, such as Compound Discoverer, MS DIALS, XCMS, and MZmine [8,24,25,26].

For each molecule the choice of the best fragmentation energy for both positive and negative polarities allowed us to optimize the identification process. Usually in discovery experiments a stepped energy of 30, 40, 50 eV is used. Our data suggest, however, that for many molecules these energies are not optimal and this could affect the correct identification of the metabolites, especially in complex matrices such as plasma and urine [27].

Our protocol was successfully applied to the two most commonly analyzed biological fluids (urine and plasma) allowing us to obtain a high number of level 1 identified metabolites: 132 in plasma and 124 in urine.

Notably, some key metabolites could be identified. Among them carnitine is particularly interesting for its involvement in brain degeneration and cognitive performance processes [28] and correlation to birth weight in healthy neonates [29].

Moreover, the presence of metabolic substrates and intermediates, both in blood and urine samples, may derive from exosomes and macrovesicles that possess a metabolic activity as previously demonstrated [30,31].

Another interesting finding is represented by the identification of metabolites associated with some diet components or a particular diet. Indeed, it was shown that diet, gut microbiome, and metabolome are closely related, with short-term diet being more strongly related to plasma metabolome [32].

Metabolites produced by the microbiome, especially in response to diet, have the potential to affect the metabolic processes of the host, both positively and negatively. The interplay between human and microbiome metabolic pathways was shown to particularly affect serum indole-containing molecules [18]. Consistently, in our plasma and urine samples, we have found several indole-containing metabolites of tryptophan. A correlation between dysbiotic microbiota and diseases, such as cancer, inflammatory bowel diseases and neurodegenerations, such as Parkinson disease [33], imply a promising role for untargeted large-scale metabolomic studies to be applied clinically.

In conclusion, in this paper we have shared a standardized workflow for achieving safe metabolite assignment by high-resolution mass spectrometry coupled with UHPLC. This information and related data can be used as a starting point by those who want to approach untargeted metabolomics analysis.

The reliable identification of several metabolites in a single chromatographic run opens interesting scenarios for the study of metabolic processes in both disease and health, quantifying markers typical of disease but also of diet—lifestyle or gut microbiome. Our aim is to apply the AMRT database on real samples in order to improve the stratification of pediatric patients, to produce new knowledge of pathology by identifying and characterizing metabolic dysregulation.

## 4. Materials and Methods

### 4.1. Chemicals

Ammonium formate, ammonium bicarbonate, acetonitrile (ACN), methanol (MeOH) and formic acid (FA), all LC-MS grade, were purchased from Sigma Aldrich Srl (Milan, Italy). Water was purified by reverse osmosis and filtrated through a Milli-Q purification system (Millipore, Milford, MA, USA).

Four reversed phase columns: ACQUITY BEH C18 (1.7 µm 2.1 × 100 mm, Waters S.p.A., Sesto San Giovanni, Milan, Italy), Hypersil GOLD aQ (2.6 µm 2.1 × 100 mm, ThermoFisher Scientific, Milan, Italy), Hypersil GOLD CN (1.9 µm 2.1 × 100, ThermoFisher Scientific, Milan, Italy), Accucore Polar Premium (2.6 µm 2.1 × 150, ThermoFisher Scientific, Milan, Italy) and four HILIC columns: ACQUITY BEH Amide (1.7 µm 2.1 × 150 mm, Waters S.p.A., Sesto San Giovanni, Milan, Italy), Accucore Amide HILIC (2.6 µm 2.1 × 150, ThermoFisher Scientific, Milan, Italy), SeQuant ZIC-pHILIC (5 µm 2.1 × 150 mm, Merck S.p.a., Milan, Italy), Shodex Asahipak NH2P-50 2D (5 µm 2.0 × 150, Phenomenex, Bologna, Italy) were employed for the development of chromatographic conditions.

### 4.2. Library of Standards

The library of standards was purchased from IROA Technologies (Bolton, MA, USA).

MSMLS contains over 634 unique small molecule compounds, provided at 5 µg per well, arranged in 7 polypropylene plates with alphanumeric assigned positions. Forty-two compounds were excluded from analyses: 5 of them were out of our defined mass range (70–1000 *m*/*z*) and 37 were duplicates. Compounds were dissolved using two different solutions (10% methanol for plates 1–5 and chloroform: methanol: water 1:1:0.3 for plates 6–7) in order to obtain a 0.1 µg/µL concentration.

### 4.3. Urine and Plasma Samples

Plasma and urine samples were obtained from 50 pediatric subjects (age 0–16 years, 25 males and 25 females) admitted at different wards of the Giannina Gaslini Institute (Genoa, Italy), a tertiary care pediatric Hospital. Leftover samples after routine analyses were used. Plasma was obtained from peripheral venous blood collected in 3 mL EDTA K3-containing tubes, centrifuged at 4000× *g* for 5 min at 4 °C. Urine was collected from single spot. A sample pool for each matrix was obtained, aliquoted and stored at −20 °C until used.

A written consent allowing the collection of leftover samples and the use of clinical and nongenetic data for clinical research was signed by patient’s guardians. No formal approval from the Internal Review Board was required since no additional blood sampling was needed in order to set up our metabolomic protocol.

A 50 µL aliquot of each pool was extracted adding 150 µL cold (−20 °C) methanol, vortex-mixed and centrifugated at 14,000 rpm for 10 min. The supernatant was collected and stored at −80 °C until analyzed.

### 4.4. Mass Spectrometric Conditions and Spectral Library

LC-HRMS analysis was carried out using a Vanquish Horizon UHPLC system coupled to a Q-Exactive Plus Hybrid Quadrupole-Orbitrap Mass Spectrometer (Thermo Fisher Scientific, Milan, Italy). Ionization was obtained using a heated electrospray source (HESI) probe both in positive and negative mode. In order to build the MSMLS spectral library, the 592 compounds contained in the corresponding wells were analyzed by FIA at a flow rate of 0.15 mL/min of 60/40 ACN/H20, 0.2% FA, and a run time of 0.5 min.

For each compound the molecular ion was selected and fragmented using 12 different collision energies from 10 to 120 eV, with steps of 10 eV. For some compounds that were not capable of generating the molecular ion in the ion source, the most abundant adduct was considered.

The following parameters were used in order to maximize signal and optimize stability (CV < 5%) for both polarities: sheath gas of 30 a.u, auxiliary gas of 10 a.u, auxiliary gas temperature of 300 °C and capillary temperature of 300 °C. A spray voltage of 4000 and 3000 V in positive and negative modes, respectively, was used. Data were acquired in MS^1^ Full Scan mode with a resolution of 70,000, automatic gain control (AGC) 1 × 10^6^, mass range 70–1000 *m*/*z* and a maximum injection time of 200 ms, followed by a parallel reaction monitoring (PRM) event with an inclusion list of only one precursor with a MS^2^ resolution of 17,500, AGC 5 ×10^5^, maximum injection time of 60 ms and an isolation window of 1.2 *m*/*z*.

All raw data file were processed with mzVault 2.1 (Thermo Fisher Scientific, Milan, Italy) to build the fragmentation library. The best energy was automatically selected by the software’s algorithm and then the results were manually reviewed ensuring that the spectra contained both the molecular ion and a sufficient number of fragments.

### 4.5. Selection of Chromatographic Conditions and Retention Time Assignment

Eight different chromatographic columns and different mobile phases were tested using a single mixture containing all the metabolites spiked in plasma. Raw data were analyzed in order to choose column and eluents which ensured the best separation using Tracefinder 4.1 software (Thermo Fisher Scientific, Milan, Italy) adopting a screening approach. In particular, for each metabolite in the library, the software extracted a mass trace, integrated the peaks found and chose the peak with the highest intensity on which the following chromatographic suitability criteria were tested: symmetry, peak width, tailing and column overload. The peaks with best scores in terms of symmetry (at peak height 50%, a threshold above 90%) and peak width (at peak height 50%, a value between 1.8 and 3.6 s) were selected.

The mobile phases tested for C18 columns were: phase A, water 0.1% FA; phase B, MeOH 0.1% FA or ACN 0.1% FA. The mobile phases tested for HILIC columns were: phase A, H_2_O 5 mM ammonium formate (pH 3) and H_2_O 5 mM ammonium bicarbonate (pH 8); phase B, ACN. The linear gradient for reversed phase columns started with 1% B and in 15 min increased up to 100% with a flow rate of 250 µL/min, then the columns were normalized for 5 min with 1% phase B. The linear gradient for HILIC columns started with 90% B and decreased to 30% B in 15 min with a flow rate of 200 µL/min, the columns were then normalized with 90% phase B for 9 min. The column temperature was maintained at 40 °C for C18 columns and at 25 °C for HILIC columns. Subsequently, to determine the retention times, MSMLS was divided into 9 mixtures composed of non-isobaric molecules (as specified in Supplementary Table S2). The 9 different mixtures were then spiked in plasma matrix, extracted and injected with the conditions selected above.

The experiments were performed in data-dependent acquisition mode (DDA) with inclusion mass list priority, with both positive and negative polarity. A maximum of 5 MS/MS experiments were triggered for each DDA scan. The intensity threshold was set at 1.6 × 10^5^ using an isolation window of 1.4 Da. The *m*/*z* values of signals already selected for MS/MS were put on an exclusion list for 20 s. Resolution of 70,000 (at *m*/*z* 200) and 17,500, AGC of 3 × 10^6^ and 1 × 10^5^, 100 ms and 50 maximum injection time were used for MS^1^ and MS^2^ scans, respectively.

### 4.6. Untargeted Metabolomic Analysis of Real Samples

Plasma and urine samples were analyzed using the chromatographic conditions which yielded the best results and analyzed in DDA mode with fragmentation priority. We have divided all the molecules of the library, based on the retention times (with a retention time window of ±0.5 min), in numerous inclusion list files limiting overlaps as much as possible. The experiments were performed separately for each polarity, alternating MS and MS/MS experiments. Resolutions of 70,000 (at *m*/*z* 200) and 17,500, AGC of 1 × 10^6^ and 2 × 10^5^, injection times of 100 and 65 ms maximum were used for MS^1^ and MS^2^ scans, respectively. The loop count was set at 5, the *m*/*z* values of the signals already selected for MS/MS were put in an exclusion list for 5 s. The minimum AGC target for the triggering of MS/MS was set at 2 × 10^3^ using an isolation window of 1.4. A normalized stepped collision energy of 20, 40, 80 was used.

For data processing, compound Discoverer ver. 3.1 (Thermo Fisher Scientific, Milan, Italy) was used, the workflow of data processing was shared (Workflow.cdProcessingWF). AMRT was used as the unique database for identification of unknown features.

## Figures and Tables

**Table 1:** Chromatographic columns and mobile phases tested during method development with the reports of how many peaks fulfill chromatographic suitability criteria: symmetry, peak width and the conjunction of both.

Column		Positive			Negative		
Reversed Phase	Phase B—Solvent	Symmetry	Width	Symmetry + Width	Symmetry	Width	Symmetry + Width
ACQUITY BEH C18	ACN	112	119	50	136	100	67
	MeOH	72	129	29	91	101	40
Hypersil GOLD CN	ACN	62	108	14	63	75	24
	MeOH	56	99	12	59	59	12
Hypersil GOLD aQ	ACN	82	137	42	110	82	49
	MeOH	60	125	19	76	60	24
Accucore Polar Premium	ACN	84	120	30	105	56	28
	MeOH	64	108	19	92	76	26
**HILIC**	**Phase A—pH**	**Symmetry**	**Width**	**Symmetry + Width**	**Symmetry**	**Width**	**Symmetry + Width**
ACQUITY BEH Amide	pH 2	75	120	25	63	72	25
	pH 8	87	133	26	38	77	21
Accucore Amide HILIC	pH 2	73	114	22	20	34	6
	pH 8	76	118	32	21	42	15
Shodex Asahipak NH2P-50 2D	pH 2	51	100	10	27	84	5
	pH 8	71	116	32	45	116	8
SeQuant ZIC-pHILIC	pH 2	65	130	18	59	60	24
	pH 8	69	120	22	38	44	18

## Data Availability

The data presented in this study are available on request from the corresponding author.

## Supplementary Materials

The following are available online. IRCCS Istituto Giannina Gaslini-Mass Spectra Library POSITIVE.msp, IRCCS Istituto Giannina Gaslini-Mass Spectra Library NEGATIVE.msp, Supplementary Table S1.xlsx, Supplementary Table S2.xlsx, Supplementary Table S3.xlsx and Workflow.cdProcessingWF.

## Sample Availability

Samples of the compounds are not available from the authors.
